# Comparison of vocal cord view between neutral and sniffing position during orotracheal intubation using fiberoptic bronchoscope: a prospective, randomized cross over study

**DOI:** 10.1186/s12871-018-0671-9

**Published:** 2019-01-05

**Authors:** Sanghee Park, Hyung Gon Lee, Jeong Il Choi, Seongheon Lee, Eun-A Jang, Hong-Beom Bae, Jeeyun Rhee, Hyung Chae Yang, Seongtae Jeong

**Affiliations:** 1 Department of Anesthesiology and Pain Medicine, Chonnam National University Medical School, Chonnam National University Hospital, 42 Jebong-ro, Dong-gu, Gwangju, 61469 South Korea; 2 Department of Otolaryngology-Head and Neck Surgery, Chonnam National University Medical School, Chonnam National University Hospital, Gwangju, South Korea

**Keywords:** Airway management, Fiberoptic bronchoscope, Intubation, Patient positioning

## Abstract

**Background:**

In intubation using fiberoptic bronchoscope (FOB), partial or complete obstruction of upper airway makes the FOB insertion difficult. Thus, maneuvers to relieve such obstructions are recommended. There have been no studies to determine whether the sniffing or neutral position is superior for this purpose. Therefore, this study was performed to examine the effects of these two positions including vocal cord view.

**Methods:**

Fifty-four patients scheduled to receive general anesthesia by orotracheal intubation were eligible for inclusion in the study with informed consent. After confirmation of proper head positioning depending on the group, the view of the vocal cord was acquired in each position. Images were reviewed using the percentage of glottic opening (POGO) score.

**Results:**

A total of 106 images of vocal cords from 53 patients were obtained. The mean of difference of POGO score was 11.09, higher for the neutral position and standard deviation was 23.73 (*p* = 0.002). Neutral position increased POGO score in 31 patients and decreased POGO score in 13 patients compare to sniffing position (*p* = 0.017). There were no significant differences between the two head positions with regard to intubation time or degree of convenience during intubation.

**Conclusions:**

Neutral position improved the view of glottic opening than sniffing position during oral fiberoptic intubation. However, there was no difference in the difficulty of tube insertion between the two positions.

**Trial registration:**

Clinical Trials.gov identifier: NCT02931019, registered on October 12, 2016.

## Background

In intubation using fiberoptic bronchoscope (FOB), partial or complete obstruction of upper airway makes the FOB insertion difficult [1], and many methods to relieve the obstruction have been reported. As FOB has become a strategic tool for endotracheal intubation [2, 3], efficient positions for fiberoptic endotracheal intubation including patient head position, have been studied [4–8].

In the sedated state, the soft palate, tongue base, and epiglottis move posteriorly, thereby obstructing the airway patency [9, 10]. A number of positions including head tilt, jaw thrust, or lingual traction have been recommended for fiberoptic intubation to relieve the obstruction at these three points [4, 5, 11]. These positions move the oropharyngeal structures anteriorly with the mouth open and an empty oropharyngeal airspace, thus enabling advancement of the FOB. However, there have been no studies to determine whether the sniffing or neutral position is superior for this purpose. Although the sniffing position has been recommended for laryngoscopic endotracheal intubation, its clinical benefit is controversial, and it is unclear whether this position is suitable for fiberoptic intubation [12–14].

In intubation using FOB, a previous endoscopic study indicated that the most common point where endotracheal tube advancement is blocked is the right arytenoid process [15]. Furthermore, the head position with neck flexion using a 7 cm high pillow aggravates the epiglottic lift and can induce the bumps between the endotracheal tube and the arytenoid process [16]. From these consequences, we deduced that the neck flexion would obstruct the advancement of endotracheal tube with limiting the view of FOB during orotracheal intubation. Therefore, we hypothesized the neutral position give better vocal cord view and ease of intubation than the sniffing position during fiberoptic orotracheal intubation.

## Methods

This prospective randomized crossover trial was approved by the Institutional Review Board of Chonnam National University Hwasun Hospital (CNUHH-2016-120) and was registered in the ClinicalTrials.gov public registry (NCT02931019). The study was conducted at the Department of Anesthesiology of Chonnam National University Hwasun Hospital, Republic of Korea, between 13 October 2016 and 2 December 2016.

### Patient selection and enrolment

Patients aged 19 to 70 years, with an American Society of Anesthesiologists classification of I–II, who were scheduled to receive general anesthesia for surgery of the thyroid, breast, stomach, colon, and uterus with orotracheal intubation, were eligible for the study. To minimize the effects of airway variation, a preoperative airway evaluation was conducted the day before surgery using the total airway score (TAS) including the Mallampati classification, thyromental distance, head and neck movement, body mass index, buck teeth, inter-incisor gap, and upper lip bite test [17]. Patients with a total airway score > 6 points or determined to be class III in the upper lip bite test were defined as being difficult to intubate and were thus excluded. Patients with a history of difficult intubation, cervical spine defect, previous head and neck surgery, or risk of pulmonary aspiration were also excluded. After the examination of 65 patients, 54 qualified for study participation and provided written informed consent.

### Study design and data collection

The patients were divided into two groups according to the order of head position: S-N group, the sniffing (S) position followed by the neutral (N) position; and the N-S group, the neutral position followed by the sniffing position. The sequence of positions was randomized by a computer-generated list of random numbers. For the neutral position, patients were placed on the operating table in the supine position without a pillow, and for the sniffing position they were kept in the supine position with a 7 cm high pillow placed under the occiput. Upon arrival in the operating room, all patients were monitored with an electrocardiogram, pulse oximeter, non-invasive blood pressure unit (Aisys; GE Healthcare, Waukesha, WI, USA) and bispectral index score (BIS; Medtronic-Covidien, Dublin, Ireland). Patients were given 0.1 mg glycopyrrolate intravenously to suppress salivation, and anesthesia was induced with 2 mg kg^− 1^ propofol followed first by remifentanil infusion of up to 3 ng mL^− 1^ using a target-controlled infusion device (Orchestra Base Primea; Fresenius Kabi, Brezins, France), and then by 0.8 mg kg^− 1^ rocuronium. Manual mask ventilation was provided until the patient’s oxygen saturation reached 100% as determined by pulse oximetry. Neuromuscular monitoring was applied with train-of-four (TOF) monitoring (Aisys; GE Healthcare). At a TOF count of 0, an Ovassapian airway (Ovassapian Fiber Optic Intubating Airway; Teleflex, Wayne, PA, USA) was inserted to guide the scope insertion through the midline of the tongue base [18, 19]. Then a specific trained anesthesia nurse conducted the jaw thrust with mouth opening, grasping patient’s both mandible angles and protruding the lower teeth until the FOB took a picture of tracheal inlet. All patients were intubated by experienced anesthesiologists using a FOB (MAF-TM Portable Bronchoscope; Olympus, Tokyo, Japan). Under the guidance of the Ovassapian airway, the FOB was inserted until just passing the tongue base and adjusted to get the best view of vocal cord with the epiglottis was acquired. At this point, the glottic opening formed by the epiglottis, vocal cord, and arytenoid cartilage was imaged (vocal cord view). Second images were taken after changing the position according to the patient group, and the FOB was passed into the trachea until it was located just above the carina. With the FOB position maintained, the Ovassapian airway was removed and the endotracheal tube (ETT) advanced smoothly into the trachea. If resistance was detected during passage into the airway, the ETT was withdrawn, rotated 90° anticlockwise, and re-inserted. The time from acquisition of the second image until completion of endotracheal intubation and the number of re-insertion trials were recorded. After proper placement of the ETT above the carina was confirmed by fiberoptic bronchoscopy view, the FOB was removed, and the patient was administered routine anesthetic care. Hypoxia, defined as oxygen saturation (SpO_2_) < 93%, and the lowest SpO_2_ value during the study were documented. The procedure was terminated if any adverse events occurred. The epiglottic view of each position was assessed by the percentage of glottic opening (POGO) score to evaluate the practitioner’s first vision of glottic opening during fiberoptic intubation [6, 20].

### Sample size justification and statistical analyses

The POGO score was used to compare the visual range of tracheal inlet during fiberoptic intubation. The sample size calculation for the present study was based on changes in POGO score between two head positions (paired t-test). According to pilot study (*n* = 12), the difference of POGO scores showed normal distribution by Kolmogorov-Smirnov test, we obtained a mean of difference = 13.80, SD of difference = 37.86 (effect size = 0.3644069) for paired t-test. Thus, 48 patients yielded a power of 80% and a type I error of 0.05. Assuming a dropout rate, 54 patients were needed for the study. The patients were allocated to the S-N or N-S group using a randomization table. POGO scores were assigned by three other anesthesiologists who were blinded to the head position group. Numerical data, including the POGO score of vocal cord images, were analyzed using the paired *t*-test because it follows the normal distribution by the Kolmogorov-Smirnov test. Changes in the number of patients in POGO score between positions were analyzed using McNemar test. Time to completion of intubation and the difficulty of tube insertion were analyzed using the Mann–Whitney U test. The relationship between difficulty of tube insertion and time to intubation was assessed by Pearson’s correlation coefficient. Data are expressed as mean ± standard deviation or as numerical values. In all of the analyses, *P* < 0.05 was taken to indicate statistical significance.

## Results

**Fig. 1:**
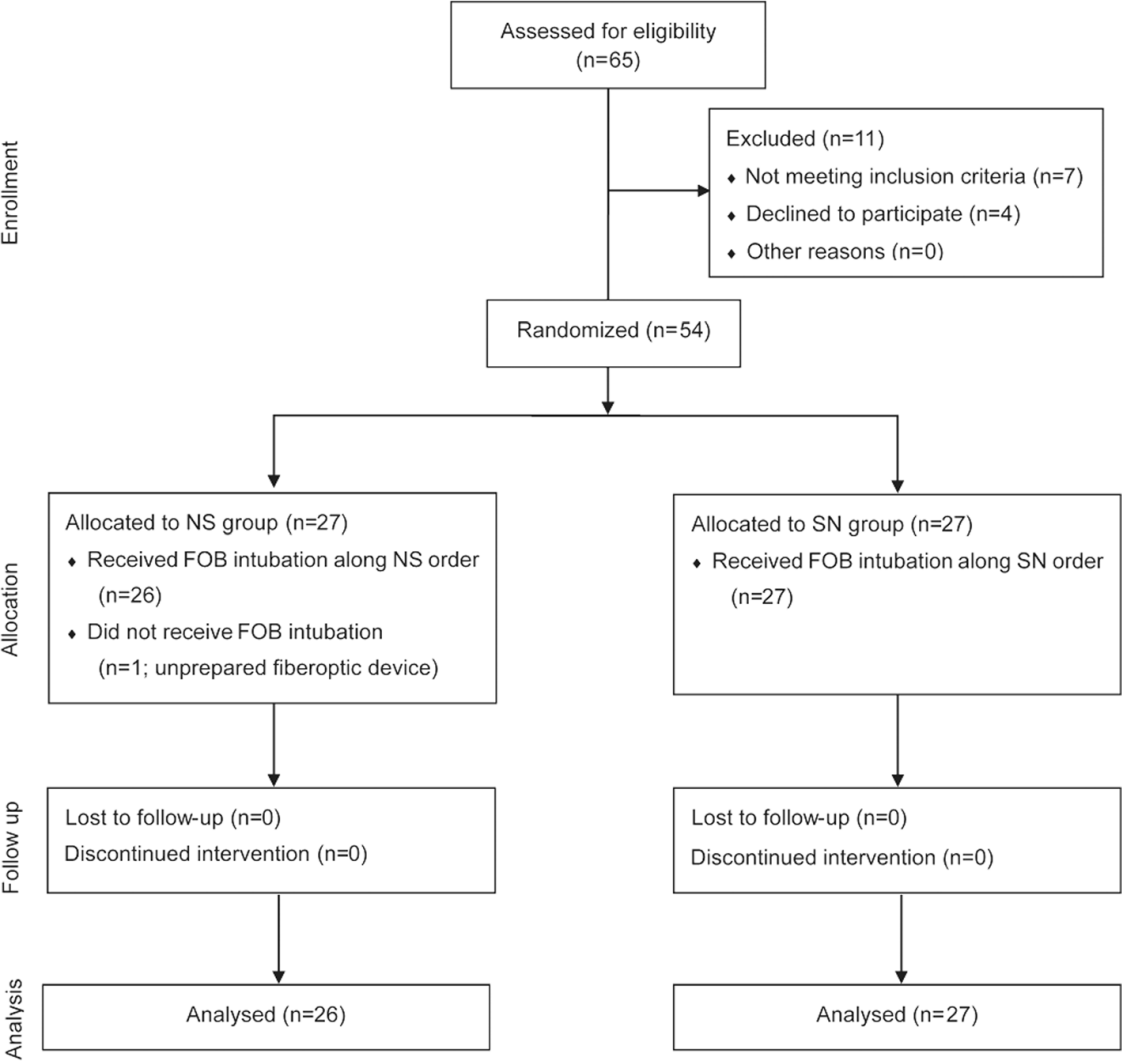
CONSORT flowchart showing the flow of patients through the trial. Group NS: Neutral position followed by sniffing position, Group SN: Sniffing position followed by neutral position, FOB = fiberoptic bronchoscope

Sixty-five patients were initially enrolled in this study, and 11 were excluded. Detailed information on patients including those who were excluded is provided in Fig. 1. The characteristics of these patients are listed in Table 1. The lowest SpO_2_ reading by pulse oximetry was 94%, recorded in one patient in the N-S group. No adverse events were observed in either group, nor were there significant differences in the hemodynamic parameters between the two groups during the study period.

**Table 1:** Characteristics of the subjects and results of preoperative airway evaluation

Characteristic	Group NS	Group SN
Subjects (*n* = 53)	26	27
Age [yr: mean (range)]	53 (32–69)	52 (38–70)
Height [cm: mean (SD)]	161 (7.8)	159 (8.1)
Weight [kg: mean (SD)]	65 (8.9)	63 (13.1)
BMI [kg m^−2^: mean (SD)]	25 (2.8)	25 (3.6)
Preoperative airway evaluation		
Mallampati class		
Class I	20	19
Class II	6	7
Class III-IV	0	1
ULBT		
Class I	21	23
Class II	5	3
Class III	0	1
Total airway score [mean (SD)]	1.15 (1.02)	1.22 (1.53)

*Group NS* Neutral position followed by sniffing position, *Group SN* Sniffing position followed by neutral position

**Fig. 2:**
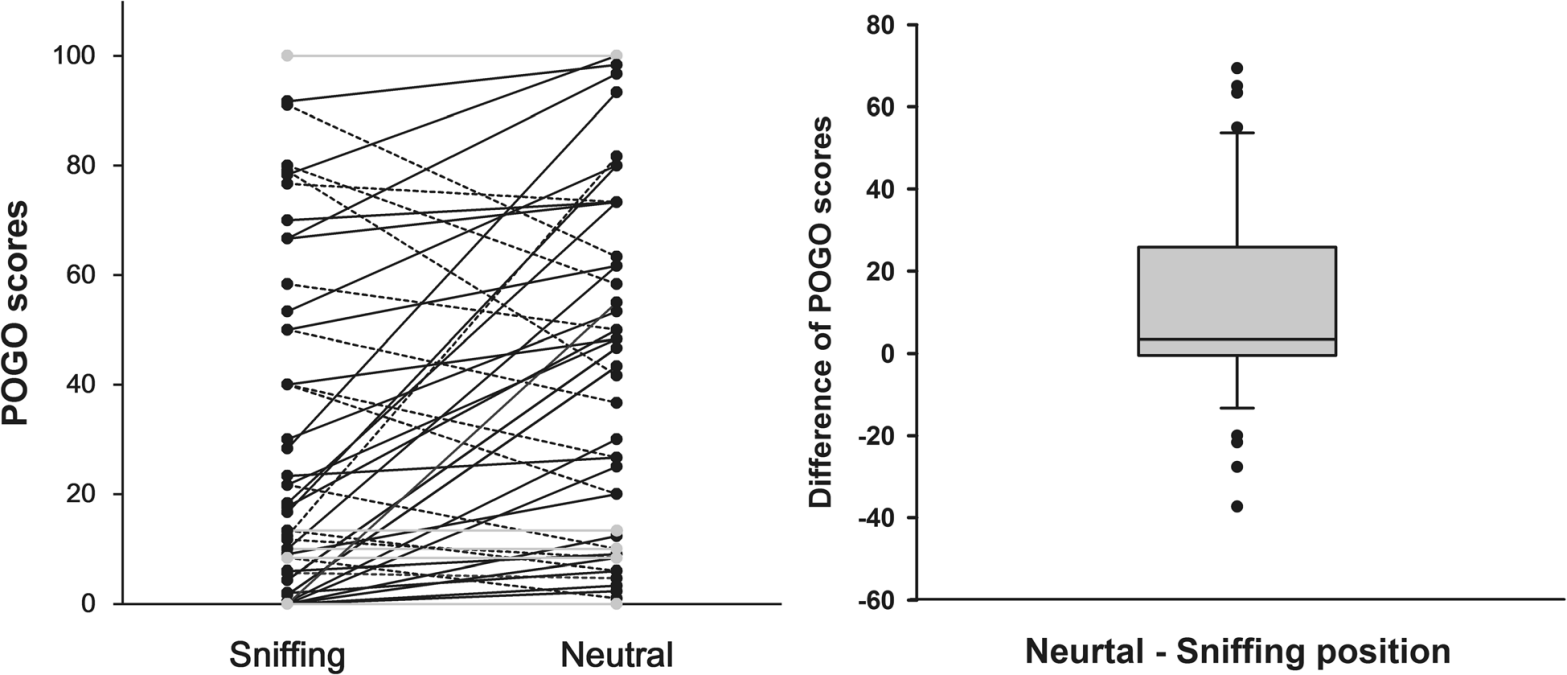
Comparison of the POGO (percentage of glottic opening) scores. A Solid line (—): Neutral position increased POGO score compare to Sniffing position (*n* = 31); a dot line (−-): decreased POGO score (*n* = 13); a grey line: no changes in POGO score (*n* = 9). The mean of difference of POGO score was 11.09, higher for the neutral position and standard deviation was 23.73 (*p* = 0.002). Neutral position significantly increased the number of patients with increased POGO score compare to sniffing position (*p* = 0.017)

As this study had a crossover design and images of each head position from 53 patients were taken, 106 images (53 images for sniffing position and 53 images for neutral position) of the vocal cord view were obtained. The mean of difference of POGO score was 11.09, higher for the neutral position and standard deviation was 23.73 (*p* = 0.002) (Fig. 2). Neutral position increased POGO score in 31 patients and decreased POGO score in 13 patients compare to sniffing position (*p* = 0.017) (Fig. 2). There were no significant differences in blockade during endotracheal tube insertion based on the number of repositioning events or handling of the FOB (Table 2) [21] between the two groups (*p* = 0.355). The times from obtaining images to completion of intubation were 23.3 ± 6.5 s in the sniffing position and 23.4 ± 11.5 s in the neutral position (*p* = 0.626). POGO score and time to completion of intubation showed a correlation of - 0.118 (*p* = 0.403).

**Table 2:** Grading for difficulty of tube insertion (DTI) and the result

Grade	Difficulty	
0	No hold-up encountered	
1	Hold-up on initial attempt, relieved by withdrawal and rotation of tube 90° anticlockwise.	
2	Hold-up on initial attempt requiring more than one manipulation of the tube, alteration in head or neck position or external manipulation	
Position	DTI Grading	Number of patients
Sniffing	0	22
	1	3
	2	1
Neutral	0	25
	1	1
	2	1

## Discussion

**Fig. 3:**
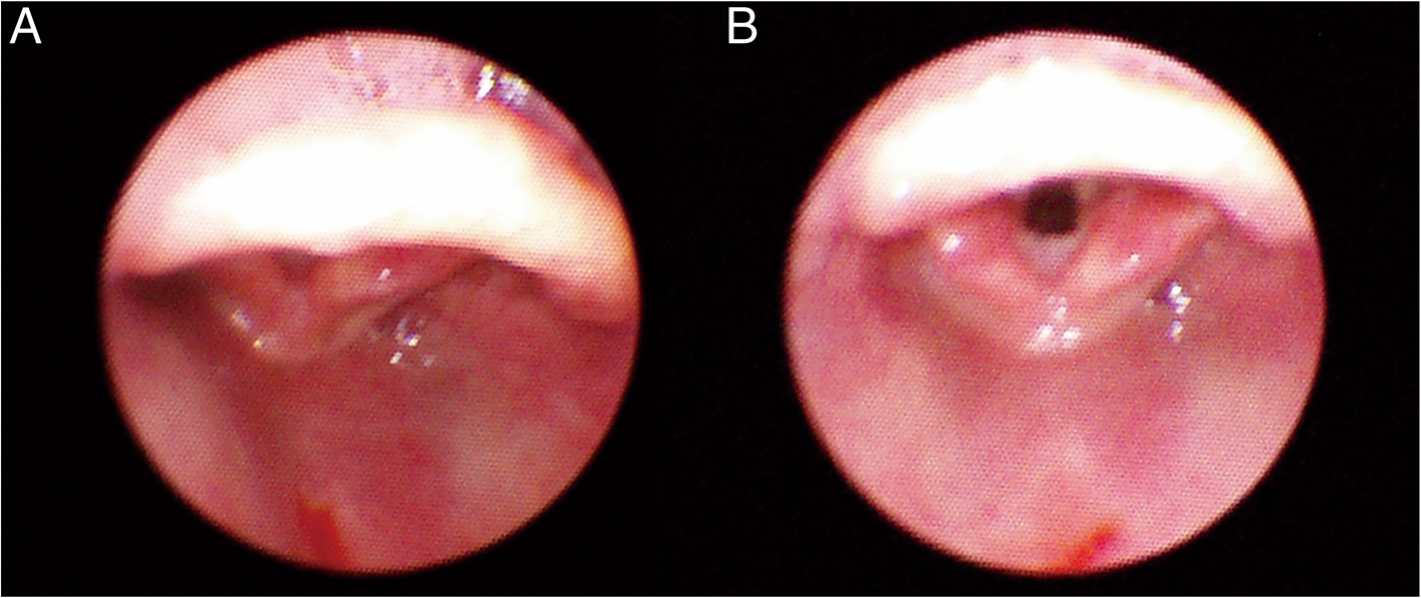
Fiberoptic bronchoscopy views of vocal cord during (**a**) sniffing position and (**b**) neutral position

In the present study, the POGO score indicating the non-obstructing area of glottic opening was significantly higher in the neutral position than in the sniffing position. This difference could be attributed to the anterior aspect of the vocal cord, which is covered by the epiglottis (Fig. 3). In the sniffing position, because the epiglottis is closer to the posterior pharyngeal wall [22] due to the stretched and collapsed retropharyngeal muscles at the neck flexion point, the area of vocal cord obscured by the epiglottis is wider than in the neutral position. In sedated patients, this tendency is stronger because of the reduced muscle tone of the retropharyngeal structure and soft palate [11, 23].

The sniffing position, achieved using a pillow, was recommended by Magill in 1936 as being better than the neutral position during endotracheal intubation using laryngoscopy [24]. The average head height needed to obtain an optimal laryngeal view was 55 mm (range: 31–71 mm) [25]. The anatomical basis of this recommendation is the three axes (oral, pharyngeal and laryngeal) alignment theory. Bannister and Macbeth suggested that the alignment of these three axes provides a better view of the vocal cords for endotracheal intubation using a laryngoscope [26].

However, the oral axes or line of view can be ignored during endotracheal intubation using FOB, because the view inside the oral cavity can be secured and the fiberoptic endoscope passed through the patient’s mouth. Thus, only the angle between the pharyngeal and laryngeal axes is important during intubation using FOB. According to a previous MRI-based study, this angle is wider in the sniffing position as the pharyngeal axes lean posteriorly with atlanto-occipital extension [27], and poorer visualization of the vocal cord during fiberoptic endotracheal intubation is assumed.

As glottic view is one of determining factor for Fremantle score using videolaryngoscopic intubation which supplies the direct glottic view [28], the better glottic view can lead the successful intubation using FOB. In the previous studies about fiberoptic intubation, there was no consistent evaluating tool for assessment of glottis view [11, 16]. Therefore, to assess the visualization of vocal cord we used the POGO score which is invented to estimate the glottic inlet in conventional laryngoscopic intubation. A validated scoring system of the percentage of glottic opening (POGO) score is used to investigate about a degree of glottic airway narrowing [29], and has high accuracy and inter-rater reliability during videolaryngoscopic intubation [30]. For applying the POGO score to the fiberoptic view which has wide angle view, we paid attention to maintain the consistency along the pictures using ovassapian airway. Also, to compensate the inconsistency of the location taking pictures between two head positions, we selected the images viewing the widest glottic opening for scoring the POGO.

In the present study, despite the apparent difference in vocal cord view between the two positions, there were no differences in the ease of intubation and the time for the endotracheal tube insertion. These observations suggested that two different positions do not affect time or ease of orotracheal intubation using FOB. Also, while visualization of the vocal cord affects the difficulty of laryngoscopic endotracheal intubation [31], the effects of vocal cord view on duration or ease of fiberoptic intubation are unclear. The time for tube insertion and the difficulty of tube insertion showed no relationship with better vocal cord view. These observations suggested that visualization of the vocal cord does not affect time or ease of orotracheal intubation using FOB.

The present study has several limitations. First, the intubation time was not measured during whole procedure but only measured from acquiring images to completion of tube insertion. There could be the difference in the time getting the best view of glottis opening between two head position. Measurement of whole intubation time may reveal the relationship between head position and intubation time. Second, we included patients with low TAS score for reducing the airway variation of subjects, excluding patients expected difficult intubation. In patients with difficult direct laryngoscopic intubation (Cormack & Lehane grade III), elevation of the head and neck beyond the sniffing position improved visualization of glottic structures [32]. In addition, sniffing position relieves obstruction of upper airway in patients with obstructive sleep apnea indicating difficult laryngoscopic intubation [33]. These results limit the application of this study’s result to various clinical situations. To validate the superiority of different head positions during fiberoptic intubation, further studies are required with larger patient populations including various airway difficulty including measurement of the entire procedure time.

## Conclusions

Neutral position improved the view of glottic opening than sniffing position during oral fiberoptic intubation. However, there was no difference in the difficulty of tube insertion between the two positions.

## Abbreviations

BIS: Bispectral index score
ETT: Endotracheal tube
FOB: Fiberoptic bronchoscope
POGO: Percentage of glottic opening
SpO_2_: Oxygen saturation
TAS: Total airway score
TOF: Train-of-four
